# Knowledge and attitudes of primary healthcare patients regarding population-based screening for colorectal cancer

**DOI:** 10.1186/1471-2407-11-408

**Published:** 2011-09-25

**Authors:** Maria Ramos, Maria Llagostera, Magdalena Esteva, Elena Cabeza, Xavier Cantero, Manel Segarra, Maria Martín-Rabadán, Guillem Artigues, Maties Torrent, Joana Maria Taltavull, Joana Maria Vanrell, Mercè Marzo, Joan Llobera

**Affiliations:** 1 Public Health Department, Balearic Islands Health Department, Spain; 2 Costa de Ponent Primary Health Care Department, Catalonian Health Institut, Barcelona, Spain; 3 Mallorca Primary Health Care Service, Balearic Island Health Service, Spain; 4 Ibiza Health Care Service, Balearic Island Health Service, Spain; 5 Menorca Health Care Service, Balearic Island Health Service, Spain

**Keywords:** Colorectal neoplasm, population-based screening, fecal occult blood test, primary healthcare, attitude, knowledge

## Abstract

**Background:**

The aim of this study was to assess the extent of knowledge of primary health care (PHC) patients about colorectal cancer (CRC), their attitudes toward population-based screening for this disease and gender differences in these respects.

**Methods:**

A questionnaire-based survey of PHC patients in the Balearic Islands and some districts of the metropolitan area of Barcelona was conducted. Individuals between 50 and 69 years of age with no history of CRC were interviewed at their PHC centers.

**Results:**

We analyzed the results of 625 questionnaires, 58% of which were completed by women. Most patients believed that cancer diagnosis before symptom onset improved the chance of survival. More women than men knew the main symptoms of CRC. A total of 88.8% of patients reported that they would perform the fecal occult blood test (FOBT) for CRC screening if so requested by PHC doctors or nurses. If the FOBT was positive and a colonoscopy was offered, 84.9% of participants indicated that they would undergo the procedure, and no significant difference by gender was apparent. Fear of having cancer was the main reason for performance of an FOBT, and also for not performing the FOBT, especially in women. Fear of pain was the main reason for not wishing to undergo colonoscopy. Factors associated with reluctance to perform the FOBT were: ***(i) ***the idea that that many forms of cancer can be prevented by exercise and, ***(ii) ***a reluctance to undergo colonoscopy if an FOBT was positive. Factors associated with reluctance to undergo colonoscopy were: ***(i) ***residence in Barcelona, ***(ii) ***ignorance of the fact that early diagnosis of CRC is associated with better prognosis, ***(iii) ***no previous history of colonoscopy, and ***(iv) ***no intention to perform the FOBT for CRC screening.

**Conclusion:**

We identified gaps in knowledge about CRC and prevention thereof in PHC patients from the Balearic Islands and the Barcelona region of Spain. If fears about CRC screening, and CRC per se, are addressed, and if it is emphasized that CRC is preventable, participation in CRC screening programs may improve.

## Background

Colorectal cancer (CRC) is a significant health problem in developed countries, both because of its high incidence and because it is accompanied by high mortality. An epidemiological analysis of all cancers in Spain indicated that CRC had the highest incidence and the second highest mortality rate for both genders. Every year, approximately 25,600 new cases of CRC are diagnosed [1] and, in 2008, 10,604 patients died from CRC (4,630 men and 5,974 women) (INEbase). An epidemiological study indicated that the incidence of CRC in Spain is increasing, but mortality therefore is declining [2].

CRC is one of the few types of cancer for which both primary and secondary prevention are possible. With respect to secondary prevention, the evidence strongly indicates that population-based screening using the fecal occult blood test (FOBT), and colonoscopy if FOBT results are positive, reduces both the incidence of and mortality from CRC [3]. Participation of a large proportion (more than 50%) of the population in testing is crucial for the success of screening programs [4]. Thus, it is necessary to ensure widespread compliance before implementation of a CRC screening program.

The Theory of Reasoned Action indicates that intention to participate in a CRC screening program overlaps with the Theory of Planned Behavior, the most proximal determinant of participation [5,6]. Intention to participate is associated with a positive attitude toward screening, and knowledge of both CRC and cancer screening in general is an important prerequisite if a positive attitude toward CRC screening is to develop [7]. The knowledge of the general population about CRC is currently poor [7,8], and gender differences in attitudes toward CRC screening are apparent [9,10].

In Spain, a National Cancer Strategy promotes the development of population screening programs for CRC, and several regions are currently implementing such programs. No program has yet been implemented in the Balearic Islands (located in the western Mediterranean Sea) whereas, in Catalonia, after completion of a pilot study, a program will soon be extended to the entire region.

The present work is part of a more comprehensive project that aims to assess the knowledge and attitudes of primary health care (PHC) professionals [11] and patients toward CRC screening. In particular, the present study is exploratory in nature, and precedes implementation of a population-based CRC screening program in the Balearic Islands. The present work was performed during implementation of a CRC screening program in Barcelona. We assessed the extent of knowledge of PHC patients about CRC, their attitudes toward population-based screening for this disease and gender differences in these respects. A secondary objective was to identify factors that might support the use of FOBT and colonoscopy in the context of CRC population-based screening

## Methods

### Design

This was a cross-sectional descriptive study based on a survey of adult patients visiting PHCs in the Balearic Islands (which had 1,014,405 inhabitants in 2007) and in the southern metropolitan area of Barcelona (with 1,275,679 inhabitants in 2007).

### Study population

Patients 50 to 69 years of age who visited PHCs for any reason from January to June 2009 were included. Patients with a diagnosis of CRC or who had a terminal illness were excluded. In both areas, sample size was calculated assuming that 50% of PHC patients would participate in a population-based screening program. Using a confidence level of 95% and a precision of 5%, the estimated sample size was 384 patients for each area. Systematic sampling of participant nurse quotas was used. The first patient (and his/her companion) scheduled to be visited on Tuesdays and Thursdays in participant nurses' agendas were invited to participate in the study if they met inclusion criteria.

### Data collection

We developed a questionnaire based on literature review [7,8,12-15]. In December 2008, we performed a pilot study by administering the questionnaire to 20 patients in one healthcare center. As a result, the wording and/or format of some questions were/was modified. Between January and June 2009, 30 nurses in the Balearic Islands and 29 nurses in Barcelona administered the final questionnaire during patient visits. All participants signed informed consent agreements.

This study was approved by the Primary Health Care Research Committee, the Balearic Islands Ethics Committee for Clinical Research, and the Ethics Committee of the Primary Care Research Institut IDIAP Jordi Gol.

### Variables

The questionnaire explored the following variables: sociodemographics; lifestyle (tobacco consumption, daily fruit and vegetable consumption, extent of physical exercise); history of chronic health problems, intestinal polyps, and cancer; use of PHC services; knowledge about cancer and CRC; past experience with cancer screening (mammography, cytology, FOBT, colonoscopy, prostate-specific antigen [PSA] measurement, and computed tomography [CT]); attitudes toward FOBT as a CRC screening tool and toward colonoscopy if an FOBT is positive; reasons for performing or not performing an FOBT; and rationales for undergoing or not undergoing colonoscopy. With respect to variables exploring knowledge and attitudes, the possible responses were: "I agree", "I disagree", or "I do not know". Questions on performance or non-performance of FOBT or colonoscopy were posed in multiple-choice format.

### Statistical analysis

Answers to questionnaires were recorded in a in a Microsoft Access database using Teleform 4.0 for Windows. We determined the frequencies of all qualitative variables and assessed the normality of quantitative variables, the means and medians of which were calculated. All variables were explored by bivariate analysis for each gender. Next, we dichotomized the variables representing support or lack of support for FOBT and colonoscopy into two categories: "Feeling reluctant" (this category included: "No, I would not do it" and "I am not sure") and "Would support" (this category included: "Yes, I would do it"). Bivariate analysis was performed using these new variables without any change in the initial categories of the other variables. Next, two logistic regression analyses were performed; the first used support or lack of support for FOBT as the dependent variable, and the second support or lack of support for colonoscopy. In both equations, all independent variables had *p*-values of < 0.1 upon bivariate analysis. Backward logistic regression analysis was next performed. Independent variables were excluded from the model when no statistically significant relationships with the dependent variable were evident, and when the estimated coefficients did not change markedly from those yielded in the previous model employing the variable. Each new model was compared with the previous model by calculation of a likelihood ratio. SPSS version 13.0 for Windows was used for all statistical analysis.

## Results

We collected 625 completed questionnaires from 24 PHC healthcare centers in the Balearic Islands and from 36 PHC centers in Barcelona. A total of 34 patients (5.2%), 67.6% of whom were male with a mean age of 58.6 years, refused to participate. Table 1 shows the demographic characteristics of participating patients. One in three (33%) participants reported visiting a healthcare center often or very often in the previous year, 43% from time-to-time, 21% occasionally, whereas 2% had not visited a center during the previous year. Most participants reported that they had high or very high confidence in PHC doctors and nurses (78% for each question).

**Table 1:** Patient characteristics

Variables	Categories	Cases (N = 625)	Valid %	Women % (N = 361)	Men % (N = 261)
Age	50-54	123	19.7	22.4	15.7
	55-59	143	22.9	24.1	21.1
	60-64	177	28.3	28.5	28.0
	65-69	182	29.1	24.9	35.2

Region	Balearic islands	254	40.6	42.2	37.2
	Barcelona	371	59.4	56.8	62.8

Educational level	< Elementary school	121	19.7	22.7	15.9
	Elementary school	385	62.7	63.2	62.0
	High school	73	11.9	9.1	15.5
	Bachelor's degree	35	5.7	5.1	6.6

Job situation	Active	242	39.0	35.5	43.2
	Not active	378	61.0	64.5	56.8

Smoking	Yes	98	15.8	12.2	20.8
	No	519	83.4	87.2	78.0

Eats fruit daily	Yes	584	93.7	93.1	94.6
	No	39	6.3	6.9	5.4

Eats vegetables daily	Yes	549	88.3	93.1	94.6
	No	73	11.7	10.0	13.8

Practices physical activity Daily	Yes	486	78.3	76.3	81.2
	No	134	21.6	23.7	18.5

Chronic health problem	Yes	452	77.7	77.1	78.6
	No	123	21.1	21.7	20.2
	Don't know	7	1.2	1.2	1.2

Type of chronic health problem	Hypertension	330	52.8	52.1	54.4
	Diabetes	175	28.0	22.4	35.6
	Depression	79	12.6	17.5	6.1
	Anxiety	66	10.6	13.9	6.1
	Heart failure	32	5.1	3.0	8.0
	Renal failure	14	2.2	1.4	3.4
	Asthma	27	4.3	4.2	4.2
	COPD	22	3.5	1.7	6.1
	Irritable bowel	16	2.6	3.0	1.1
	Diverticulosis	12	1.9	2.5	1.1
	Ulcerative colitis	4	0.6	0.6	0.8

History of polyps	Yes	30	4.8	5.8	3.5
	No	567	91.3	90.9	91.8
	Don't know	24	3.9	3.3	4.7

History of cancer	Yes	62	10.1	10.4	9.8
	No	540	88.1	87.3	89.0
	Don't know	11	1.8	2.3	1.2

Type of cancer	Breast	20	-	5.5	-
	Skin	13	2.1	1.4	3.1
	Urinary bladder	4	0.6	0.0	1.5
	Lung	2	0.3	0.3	0.4
	Prostate	8	-	-	3.1
	Other	11	1.8	1.4	2.3

Family history of colorectal cancer	Yes	108	17.5	21.1	12.5
	No	472	77.1	74.4	80.8
	Don't know	33	5.4	4.5	6.7

Table 2 shows respondent knowledge about cancer in general and CRC in particular. Most patients knew that many cancers could be avoided by giving up smoking and that diagnosis before symptom occurrence improved the chance of survival. However, only half of all respondents knew that more than 50% of CRC patients survive for 5 years after diagnosis or that exercise could help prevent CRC. It was also known that many cancers could be avoided by eating more fruit and vegetables and that intestinal polyps must be removed because they can become cancerous. Women had more knowledge of CRC symptoms than did men, and they were aware of the significance of bloody stools, diarrhea, and constipation, but not of other signs and symptoms, such as weight loss, tenesmus, and abdominal pain.

**Table 2:** Knowledge about cancer and colorectal cancer

Questions	Answers	Total % (N = 625)	Women % (N = 361)	Men % (N = 261)	p
There are many types of cancer	Trae	94.3	95.0	93.5	0.729
	False	0.3	0.3	0.4	
	I don't know	5.3	4.8	6.2	

Some cancers can be cured	Trae	93.2	93.8	92.3	0.617
	False	3.4	2.8	4.2	
	I don't know	3.4	3.4	3.5	

Cancer is a fatal disease	Trae	27.9	27.0	29.1	0.801
	False	65.4	65.9	64.7	
	I don't know	6.7	7.1	6.2	

Many cancer cases could be avoided by doing more exercise	Trae	45.1	39.4	53.1	0.003
	False	17.1	18.7	15.0	
	I don't know	37.7	41.9	31.9	

Many cancer cases could be avoided by giving up smoking	Trae	92.2	90.2	95.0	0.065
	False	2.8	3.1	2.3	
	I don't know	5.0	6.7	2.7	

Many cancer cases could be avoided by eating more fruits and vegetables	Trae	69.9	68.8	71.3	0.266
	False	7.5	8.9	5.4	
	I don't know	22.7	22.3	23.3	

Cancer diagnosis before symptoms can improve chances of survival	Trae	88.2	88.5	87.7	0.476
	False	1.0	0.6	1.5	
	I don't know	10.8	10.9	10.7	

More than half of colorectal cancer cases survive five years after diagnosis	Trae	44.7	45.3	43.8	0.759
	False	7.6	8.1	6.9	
	I don't know	47.7	46.6	49.2	

Intestinal polyps must be removed because they can become a cancer	Trae	64.2	66.8	60.6	0.224
	False	2.6	2.8	2.3	
	I don't know	33.2	30.4	37.1	

Which of the following symptoms indicate a colorectal cancer	Bloody stools	72.2	76.5	66.3	0.006
	Diarrhea-Constipation	42.9	48.5	35.2	0.001
	Abdominal pain	23.6	24.1	23.0	0.775
	Headache	8.8	8.0	10.0	0.475
	Fatigue	37.9	39.6	35.6	0.317
	Paleness	32.0	34.3	28.7	0.163
	Difficulty swallowing	13.8	13.9	13.8	1.000
	Weight loss	55.6	61.5	47.5	0.001
	Burning stomach	15.6	14.7	16.9	0.502
	Tenesmus	22.2	24.9	18.4	0.063
	Pain during defecation	36.2	37.1	34.9	0.612
	I don't know	20.9	17.2	26.1	0.009

A total of 82% of women and 38% of men had participated in screening tests for prevention of some type of cancer. Among women, 83.1% had undergone mammography, 68.1% cytology tests, 16.3% colonoscopies, 9.4% FOBTs, and 8.3% CT scans. Of all men, 36.4% had undergone PSA tests, 10.7% colonoscopies, 8.8% FOBTs, and 6.5% CT scans.

Patients were asked how they would respond if a PHC doctor or nurse proposed that an FOBT be performed for CRC screening. A total of 88.8% of participants reported that they would undergo the test, 7.3% were not sure, and 3.9% indicated they would not. If the FOBT was positive and a colonoscopy was offered, 84.9% of participants reported that they would undergo the procedure, 5.9% were not sure, and 9.2% would not. Responses did not differ significantly between gender.

**Figure 1:**
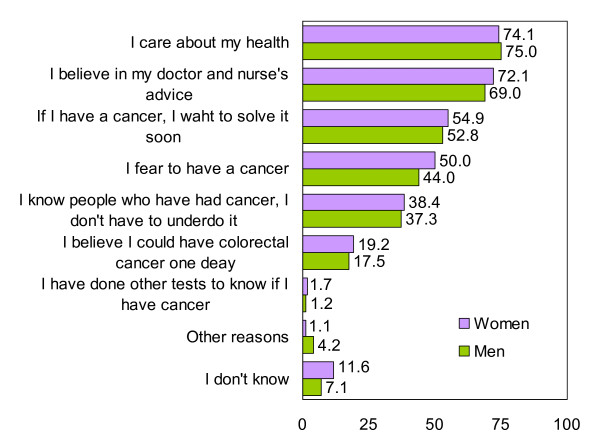
**Reasons for performing a FOBT in % (Only participants that would do it or doubt = 599)**.

**Figure 2:**
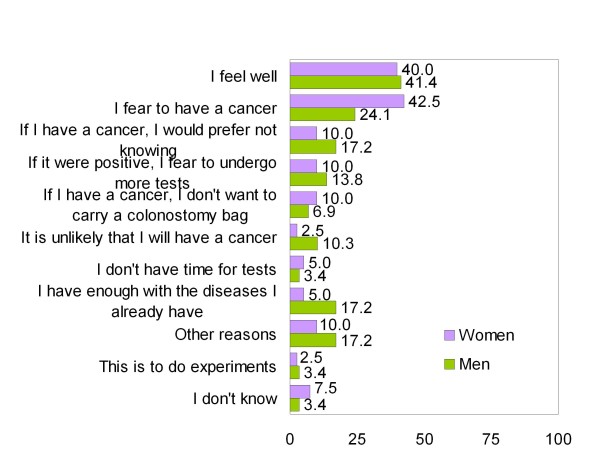
**Reasons for not performing a FOBT in % (Only participants that wouldn't perform it or doubt = 69)**.

**Figure 3:**
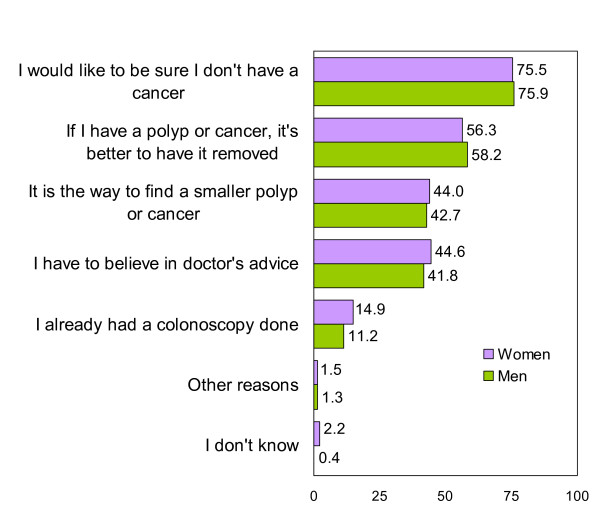
**Reasons for undergoing a colonoscopy (Only participants that would undergo it or doubt = 558)**.

**Figure 4:**
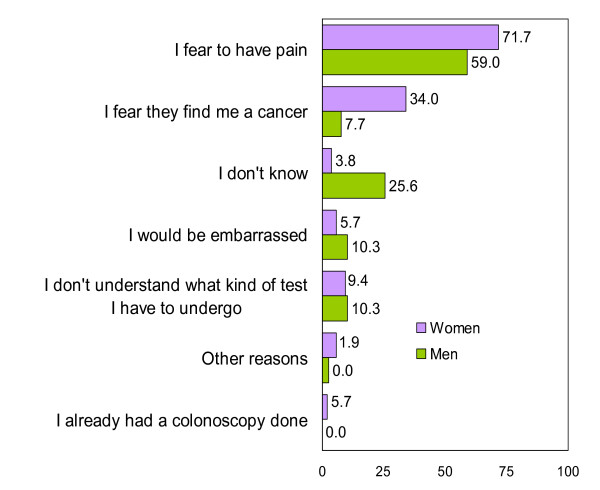
**Reasons for not undergoing colonoscopy (Only participants that wouldn't undergo it or doubt = 92)**.

Patients reported that their main reasons for performing the FOBT were that they cared about their health and that they believed in advice received from doctors and nurses (Figure 1). The main reasons why patients would not perform the FOBT were that they felt well and feared discovering cancer (Figure 2). Women reported cancer fears somewhat more frequently than did men, although the difference was not significant. Less than 20% of participants reported that they felt susceptible to CRC. The main reasons for undergoing colonoscopy were to seek reassurance that cancer was absent and the belief that, if a polyp or cancer was present, treatment was necessary (Figure 3). Fear of pain was the main reason for not undergoing colonoscopy, especially among women (Figure 4).

Bivariate analysis indicated that several factors were associated with reluctance to perform the FOBT (Table 3) and to undergo colonoscopy if the FOBT was positive (Table 4). In both instances, the knowledge that many forms of cancer can be prevented by performing more exercise and that cancer diagnosis before symptom onset can improve survival were associated with favorable views on the FOBT and colonoscopy. Knowledge of the main symptoms of colorectal cancer; experience with any screening test for cancer prevention; and a positive attitude toward colonoscopy (when FOBT was explored) or toward FOBT (when colonoscopy was explored) were the main factors associated with reluctance to undergo FOBT or colonoscopy.

**Table 3:** Bivariate analysis of factors associated (p < 0.1) with being reluctant to perform a FOBT for colorectal cancer early diagnosis

Variables	Categories		Reluctant (%)	Would support (%)	p
Job situation	Active		7.5	92.5	0.019
	Not active		13.6	86.4	

Educational level	< Elementary school		16.8	83.2	0.082
	Elementary school		10.4	89.6	
	High school		5.5	94.5	
	Bachelor's degree		14.3	85.7	

There are many types of cancer	True		10.3	89.7	0.044
	False + don't know		22.9	77.1	

Cancer is a fatal disease	True + don't know		14.2	85.8	0.080
	False		9.5	90.5	

Many cancer cases could be avoided by doing more exercise	True		5.1	94.9	0.000
	False + don't know		15.9	84.1	

Many cancer cases could be avoided by giving up smoking	True		10.0	90.0	0.013
	False + don't know		22.9	77.1	

Many cancer cases could be avoided by eating more fruits and vegetables	True		9.0	91.0	0.012
	False + don't know		16.2	83.8	

Cancer diagnosis before symptoms can improve survival	True		9.0	91.0	0.000
	False + don't know		26.8	73.2	

Intestinal polyps must be removed, because they can become cancer	True		8.4	91.6	0.010
	False + don't know		15.3	84.7	

Any screening test done for cancer prevention	Yes		8.6	91.4	0.014
	No		15.5	84.5	

PSA test done for cancer prevention	Yes		5.2	94.8	0.051
	No		12.2	87.8	

FOBT done for cancer prevention	Yes		1.8	98.2	0.014
	No		12.1	87.9	

Which of the following symptoms indicate a colorectal cancer	Bloody stools	Yes	8.4	91.6	0.001
		No	18.2	81.8	
	Diarrhea-Constipation Yes		7.4	92.6	0.010
		No	14.0	86.0	
	Abdominal pain	Yes	6.1	93.9	0.034
		No	12.7	87.3	
	Fatigue	Yes	7.6	92.4	0.035
		No	13.3	86.7	
	Weight loss	Yes	8.9	91.1	0.055
		No	13.9	86.1	
	Burning stomach	Yes	5.1	94.9	0.036
		No	12.2	87.8	
	Tenesmus	Yes	3.6	96.4	0.001
		No	13.3	86.7	
	Pain during defecation	Yes	5.3	94.7	0.000
		No	14.5	85.5	
	I don't know	Yes	18.6	81.4	0.004
		No	9.1	90.9	

In case FOBT were + and a colonoscopy were recommended, would you accept to undergo it?	Yes		5.2	94.8	0.000
	No + I doubt		44.6	55.4	

**Table 4:** Bivariate analysis of factors associated (p < 0.1) with being reluctant to undergo a colonoscopy for colorectal cancer early diagnosis

Variables	Categories		Reluctant (%)	Would support (%)	p
Region	Balearic Islands		10.0	90.0	0.004
	Barcelona		18.4	81.6	

Job situation	Active		11.8	88.2	0.082
	No active		17.2	82.8	

There are many types of cancer	True		14.2	85.8	0.028
	False + don't know		28.6	71.4	

Many cancer cases could be avoided by doing more exercise	True		10.4	89.6	0.008
	False + don't know		18.1	81.9	

Many cancer cases could be avoided by eating more fruits and vegetables	True		12.9	87.1	0.026
	False + don't know		20.1	79.9	

Cancer diagnosis before symptoms can improve chances of survival	True		12.4	87.6	0.000
	False + don't know		35.7	64.3	

More than half of cases of colorectal cancer survive 5 years after diagnosis	True		10.7	89.3	0.009
	False + don't know		18.5	81.5	

Intestinal polyps must be removed, because they can become cancer	True		12.1	87.9	0.017
	False + don't know		19.5	80.5	

Any screening test done for cancer prevention	Yes		12.7	87.3	0.067
	No		18.7	81.3	

Colonoscopy done for cancer prevention	Yes		3.4	96.6	0.001
	No		16.9	83.1	

CT done for cancer prevention	Yes		4.3	95.7	0.032
	No		15.9	84.1	

Which of the following symptoms indicate a colorectal cancer	Bloody stools	Yes	11.9	88.1	0.001
		No	23.4	76.6	
	Diarrhea-Constipation	Yes	10.9	89.1	0.012
		No	18.2	81.8	
	Abdominal pain	Yes	10.3	89.7	0.084
		No	16.5	83.5	
	Fatigue	Yes	9.4	90.6	0.002
		No	18.4	81.6	
	Paleness	Yes	10.1	89.9	0.021
		No	17.3	82.7	
	Difficulty swallowing	Yes	7.1	92.9	0.032
		No	16.2	83.8	
	Weight loss	Yes	12.0	88.0	0.022
		No	18.8	81.2	
	Burning stomach	Yes	7.2	92.8	0.019
		No	16.4	83.6	
	Tenesmus	Yes	6.5	93.5	0.001
		No	17.4	82.6	
	Pain during defecation	Yes	8.0	92.0	0.000
		No	19.0	81.0	
	I don't know	Yes	24.4	75.6	0.002
		No	12.5	87.5	

Would you accept to perform a FOBT for colorectal screening?	Yes		9.3	90.7	0.000
	No + I doubt		60.3	39.7	

Multivariate analysis indicated that patients who did not know that many cancers can be prevented by performing more exercise, and those who would not undergo colonoscopy if an FOBT was positive, were more reluctant to perform the FOBT for CRC screening (Table 5). With respect to colonoscopy, participants from Barcelona who did not know that early diagnosis of CRC was associated with improved prognosis, those who had never had colonoscopies, and those who would not perform the FOBT for CRC screening, were more reluctant to undergo colonoscopy.

**Table 5:** Multivariate analysis of factors associated with being reluctant to do a FOBT and a colonoscopy for colorectal cancer screening*

Variable	Categories	β	p	OR	95% CI
Being reluctant to perform a FOBT					

Labour situation	Active	1			
	No active	0.641	0.072	1.914	0.044-3.880
Many cancer cases could be avoided by doing more exercise	True	1			
	False + don't know	1.155	0.002	3.174	1.542-6.532
FOBT done for cancer prevention	Yes	1			
	No	2.032	0.061	7.631	0.912-63.822
Bloody stools is a symptom of colorectal cancer	Yes	1			
	No	0.617	0.066	1.853	0.960-3.579
If FOBT were positive, would you accept to undergo a colonoscopy?	Yes	1			
	No + I doubt	2.603	0.000	13.507	7.144-25.536

Being reluctant to undergo a colonoscopy					

Region	Balearic Islands	1			
	Barcelona	0.798	0.012	2.220	1.188-4.149
Cancer diagnosis before symptoms can improve chances of survival	True	1			
	False + don't know	0.822	0.023	2.276	1.117-4.635
More than half of cases of colorectal cancer survive 5 years after diagnosis	True	1			
	False + don't know	0.500	0.101	1.649	0.907-2.997
Colonoscopy done for cancer prevention	Yes	1			
	No	1.478	0.022	4.383	1.238-15.514
Fatigue is a symptom of colorectal cancer	Yes	1			
	No	0.505	0.106	1.657	0.898-3.058
Would you accept to perform a FOBT for colorectal screening?	Yes	1			
	No + I doubt	2.726	0.000	15.272	7.852-29.703

* Nagelkerke's R^2^: 0.352 for being reluctant to do a FOBT and 0.323 for being reluctant to do a colonoscopy

## Discussion

We examined the extent of knowledge about CRC in PHC patients from two regions of Spain, and the attitudes toward CRC and screening for the cancer. Our results indicate that knowledge about CRC in the general population could be improved, but that attitudes toward the FOBT and colonoscopy were generally positive. Our results also indicated some differences between men and women in attitudes toward CRC screening. This issue will be more thoroughly explored, in a qualitative manner, during the next phase of our study.

Our patients showed clear gaps in knowledge about CRC prevention and symptoms, as also reported in previous studies [7,8,14]. Women had a better knowledge of CRC symptoms and men had more knowledge of CRC prevention. A previous study in the United Kingdom also found that women had more knowledge about CRC than did men [7]. Although a general knowledge of CRC is not enough to raise CRC awareness to the level required for participation in screening programs, such knowledge has been reported as essential for development of a positive attitude toward screening programs in some studies [7,16], but not in others [17].

Most of our PHC patients (88.8%) reported that they would support a population-based screening program for CRC that employed the FOBT followed by colonoscopy in instances of FOBT-positivity. The proportion of responsive PHC patients in the United Kingdom was similar [7], but fewer patients in Japan responded positively [16]. However, an intention to undergo CRC screening is not the same as actual participation in such screening. In particular, Herbert et al. showed that whereas over 80% of participants expressed an intention to participate in a CRC screening program, only 40% actually participated [12]. Thus, it is possible that our results were influenced by social desirability bias (over-reporting of expected behavior) and by the administration of the questionnaire in healthcare centers.

One limitation of the present study is that our PHC patients may not be representative of the general population of Spain, the true target of population-based CRC screening. Spain has a free public healthcare system that covers 99% of the population. Thus, although our participants may not reflect the general population, they may be representative of those of lower socioeconomic status, and such subjects would benefit most from a campaign seeking to improve awareness of CRC screening [7].

In the present study, women reported more prior experience with cancer screening than did men. This reflects the existence of well-established screening programs for breast and cervical cancer. Thus, we expected to find differences between men and women regarding intention to participate in a CRC screening program [18], but we in fact found no gender-based difference in this variable, unlike what was noted in studies in the United Kingdom [19] and Catalonia [20], both of which reported higher participation by women in CRC screening programs.

Fear of being diagnosed with cancer, and of pain during colonoscopy, were the principal reasons given, especially by women, for not wishing to participate in CRC screening. These observations agree with those of other studies [17,21] and with the views held by PHC professionals about their patients [11]. Also, patients perceived that the risk of developing CRC was low, as has also been observed in previous studies [8]. We found no between-gender difference in perceived fear of CRC, in contrast to the results of a previous qualitative study which found that women believed that CRC was more common in men, and the women thus felt less vulnerable to this cancer [22].

Factors associated with a positive attitude toward the FOBT and colonoscopy were diverse in nature and included knowledge about CRC primary prevention, of the symptoms of CRC, and of the benefits afforded by CRC screening. Moreover, positive attitudes toward the FOBT and colonoscopy were associated, and vice versa. Previous studies also found that the perceived benefits and barriers were the main factors associated with an intention to undergo colonoscopy after a positive FOBT [16]. In one previous work, compliance with the advice of the PHC doctor was associated with intention to perform the FOBT for colorectal cancer screening, and also with actual FOBT completion [12]. Another qualitative study found that lack of trust in doctors was a barrier to CRC screening [15]. In the present work, we found no association between a positive attitude toward CRC screening and patient confidence in the PHC doctor or nurse. We suggest further exploration of this issue, because previous experience has shown that PHC doctors play key roles in developing patient willingness to participate in CRC screening [23].

Our results showed that the knowledge that physical activity could protect against CRC was associated with a positive attitude toward the FOBT. Also, we observed that an understanding that early diagnosis of CRC is associated with better prognosis was associated with a positive attitude toward colonoscopy if an FOBT was positive. It is noteworthy that one-third of our subjects did not know that polyps should be removed because they can become cancerous. Together, our results indicate that developing knowledge on CRC preventability should be a key plank in the design of an awareness program promoting CRC population-based screening, as has been noted previously [17].

## Conclusions

In summary, the present study has shown that PHC patients have knowledge gaps with respect to both the nature and prevention of CRC. Addressing patient cancer fears and emphasizing that CRC is preventable will be key elements in the successful promotion of CRC screening.

## Competing interests

The authors declare that they have no competing interests.

## Authors' contributions

MR, EC, ME, JMT, JMV, JL and GA designed the study; MR and ML led development of the projects in the Balearic Islands and Barcelona, respectively; MMR, XC, MS, and MT coordinated study work in their respective areas. MR and ME performed the statistical analysis, and MR drafted the manuscript. ME, EC, MM, MMR, XC, MS, GA, MT, JMT, JMV, JL and ML critically reviewed the draft and approved the final manuscript.

## Pre-publication history

The pre-publication history for this paper can be accessed here:

http://www.biomedcentral.com/1471-2407/11/408/prepub
